# Saponin-Based Nanoemulsification Improves the Antioxidant Properties of Vitamin A and E in AML-12 Cells

**DOI:** 10.3390/ijms17091406

**Published:** 2016-08-26

**Authors:** Qaisra Naheed Choudhry, Mi Jeong Kim, Tae Gyun Kim, Jeong Hoon Pan, Jun Ho Kim, Sung Jin Park, Jin Hyup Lee, Young Jun Kim

**Affiliations:** Department of Food and Biotechnology, Korea University, 2511 Sejongro, Jochiwon, Sejong 339-700, Korea; qaisra@korea.ac.kr (Q.N.C.); micaella76@gmail.com (M.J.K.); summermoon@korea.ac.kr (T.G.K.); jh_pan@korea.ac.kr (J.H.P.); phillip@korea.ac.kr (J.H.K.); timothypsj@hanmail.net (S.J.P.); jinhyuplee@korea.ac.kr (J.H.L.)

**Keywords:** vitamin A, vitamin E, nanoemulsification, saponin, antioxidant capacity

## Abstract

Our work aimed to investigate the protective effects of saponin-based nanoemulsions of vitamin A and E against oxidative stress-induced cellular damage in AML-12 cells. Saponin nanoemulsions of vitamin A (SAN) and vitamin E (SEN) were prepared by high-pressure homogenization and characterized in terms of size, zeta potential, and polydispersity index. SEN and SAN protect AML-12 cells against oxidative stress-induced cellular damage more efficiently via scavenging reactive oxygen species (ROS), and reducing DNA damage, protein carbonylation, and lipid peroxidation. These results provide valuable information for the development of nanoemulsion-based delivery systems that would improve the antioxidant properties of vitamin A and E.

## 1. Introduction

Oxidative stress results from an imbalance of excessive reactive oxygen species (ROS) formation and inadequate antioxidant defense system [1]; thus, it leads to certain pathological conditions [2]. ROS accumulation promotes cell death via damage to cellular macromolecules made up of lipids, proteins, and DNA [3,4]. Oxidative stress-mediated cellular damage appeared to be the main factor in the pathogenesis of a variety of degenerative diseases, including various neurodegenerative diseases, cancers, aging-associated diseases, cardiovascular disease, skin disease, and lung disease [5,6,7,8]. In addition, it is particularly associated with various liver diseases, such as obesity and autoimmune-related liver diseases [9,10].

Vitamin E is a fat-soluble vitamin [11], of which α-tocopherol is the most abundant and biologically active. α-tocopherol is a lipophilic antioxidant that strongly scavenges free radicals; it also protects lipids from oxidation, but is highly unstable [12,13]. Vitamin A is another essential vitamin [14], the deficiency of which causes stillbirth and developmental defects [15], night blindness during pregnancy [16], and increased chances of infectious disease mortality, especially in children [17,18].

Owing to the enormous potential health effects of vitamin A and E, such as prevention of inflammation-related diseases, cancer, morbidity from low birth weight [19,20,21], and oxidation [22,23], it is important to maintain the recommended intake of these vitamins. Nevertheless, the incorporation of fat-soluble vitamins into aqueous-based food has proved to be challenging, owing to their low water solubility and stability.

If these vitamins can be incorporated into aqueous-based products, the encapsulated vitamins could overcome the stability- and solubility-related issues. Various encapsulation methods for safe and stable delivery have been developed in the food industry for the incorporation of these vitamins into functional food recipes [24,25]. A variety of natural emulsifiers are being utilized for food emulsion and nanoemulsion systems [26]; this is because of the health benefits of incorporating vitamins into functional food and beverages [27]. Modern consumers prefer functional food products that have auxiliary health benefits [28].

In the current study, we focused on the utilization of saponin as a natural emulsifier as well as an antioxidant agent, which was previously reported to form a stable nanoemulsion of fat-soluble compounds. The stability of vitamin A and E nanoemulsions with saponin as an emulsifier were tested based on particle size, zeta potential, and diameter. The stable saponin emulsions of vitamins were subsequently investigated for their effectiveness against H_2_O_2_-induced oxidative stress in AML-12 hepatocytes; we evaluated whether encapsulated vitamin A and E can provide significantly higher protection against ROS-induced oxidative stress by maintaining cellular redox status and reducing oxidative damage.

## 2. Results

### 2.1. Nanoemulsion Preparation and Characterization

Nanoemulsions were prepared by high pressure homogenizer as described previously [29]. (Figure S1). Surface morphology and size are important parameters for nanoemulsion stability and interaction with biological membranes [30]. Figure S2 shows the physiochemical properties of the saponin-based vitamin nanoemulsions. The average sizes of vitamin A and E nanoemulsions were 115 nm and 120 nm, respectively. A zeta-potential value (droplet surface charge) of −25 to −30 mV is sufficient to create a high-energy barrier with the anionic molecules on the surface of the emulsion droplets, thus providing good colloidal stability [31]. The mean zeta-potential of both saponin vitamin A nanoemulsion (SAN) and saponin vitamin E nanoemulsion (SEN) was above −40 mV, thereby indicating stable emulsions with dispersed droplets in the nanometer range.

### 2.2. Cytotoxicity

The cytotoxicities of vitamin A, vitamin E, SAN, SEN, and empty saponin nanoemulsion (SN) were determined in AML-12 mouse hepatocyte cells by MTT assay, upon 16 h of exposure. The final concentration of vitamin A and E was 666.6 IU/mL and 0.18 IU/mL respectively. Equivalent concentrations of SAN, SEN and SN (20 µg/mL) were used for the comparative studies. Vitamin solution were prepared freshly every time for treatment of samples. At this concentration vitamin A, vitamin E, SAN, SEN, and SN showed no cytotoxicity in AML-12 cells after 16 h of exposure (Figure S3a). For further oxidative stress studies, we used above dose with 16 h of exposure.

### 2.3. Nanoemulsion Protection of Hepatocytes from Oxidative Stress-Induced Cytotoxicity

**Figure 1:**
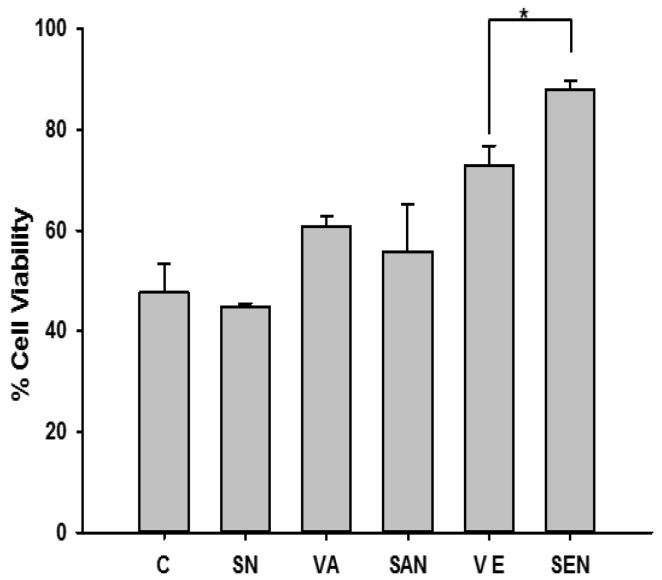
Nanoemulsion protection against H_2_O_2_-induced hepatocyte cytotoxicity. AML-12 cells were exposed to exogenous H_2_O_2_ for 16 h. Cell viability was determined by MTT assay. Vitamin and VE, vitamin E;nanoemulsion treatment protected hepatocytes against H_2_O_2_-induced cytotoxicity. C, control; SN, empty saponin nanoemulsion; VA, vitamin A; SAN, saponin nanoemulsion of vitamin A; VE, vitamin E; SEN, saponin nanoemulsion of vitamin E. All values are represented as mean ± SEM from three or more independent studies. * *p* < 0.05.

H_2_O_2_ is widely used as an oxidizing reagent to trigger oxidative stress. In our study, H_2_O_2_ was used to induce oxidative stress at various concentrations for 16 h, and cell viability was determined after 16 h of exposure. H_2_O_2_ exposure reduced cell viability in a dose-dependent manner (Figure S3b). To examine the protective effects of SAN and SEN, mouse hepatocytes were co-treated with nanoemulsions and 0.1 mM H_2_O_2_, and cell viability was measured. Results showed that SEN significantly protected (*p* < 0.05) against H_2_O_2_-induced cytotoxicity compared with vitamin E whereas A and SAN showed almost similar effect; however, SN alone did not provide a protective effect (Figure 1).

### 2.4. Cellular Antioxidant Activity (CAA) of Nanoemulsions

**Figure 2:**
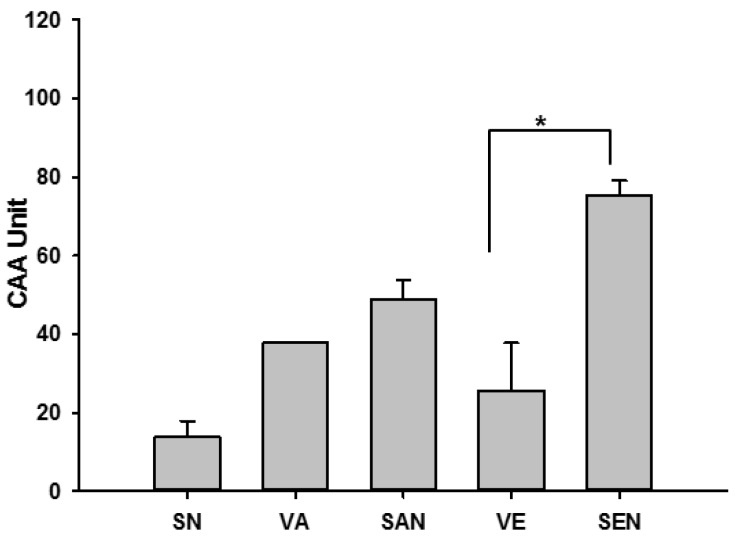
Effect of nanoemulsion on the cellular antioxidant activity (CAA) of HepG-2 cells treated with emulsified and non-emulsified samples. SN, empty saponin nanoemulsion; VA, vitamin A; SAN, saponin nanoemulsion of vitamin A; VE, vitamin E; SEN, saponin nanoemulsion of vitamin E. All values are represented as mean ± SEM from three or more independent studies. * *p* < 0.05.

In order to quantify and compare the intracellular antioxidant activity of emulsions and non-emulsions, CAA assay was performed using HepG-2 cells. A decrease in cellular fluorescence is directly proportional to the degree of oxidation [32]. Increased fluorescence from DCF-DA oxidation was inhibited by SEN, thus indicating that it had better antioxidant capacity compared with vitamin E (*p* < 0.05). On the other hand, there was no significant difference in the antioxidant capacity between vitamin A and SAN (*p* > 0.05). SN had the least antioxidant activity (Figure 2).

### 2.5. Cellular Redox Status

**Figure 3:**
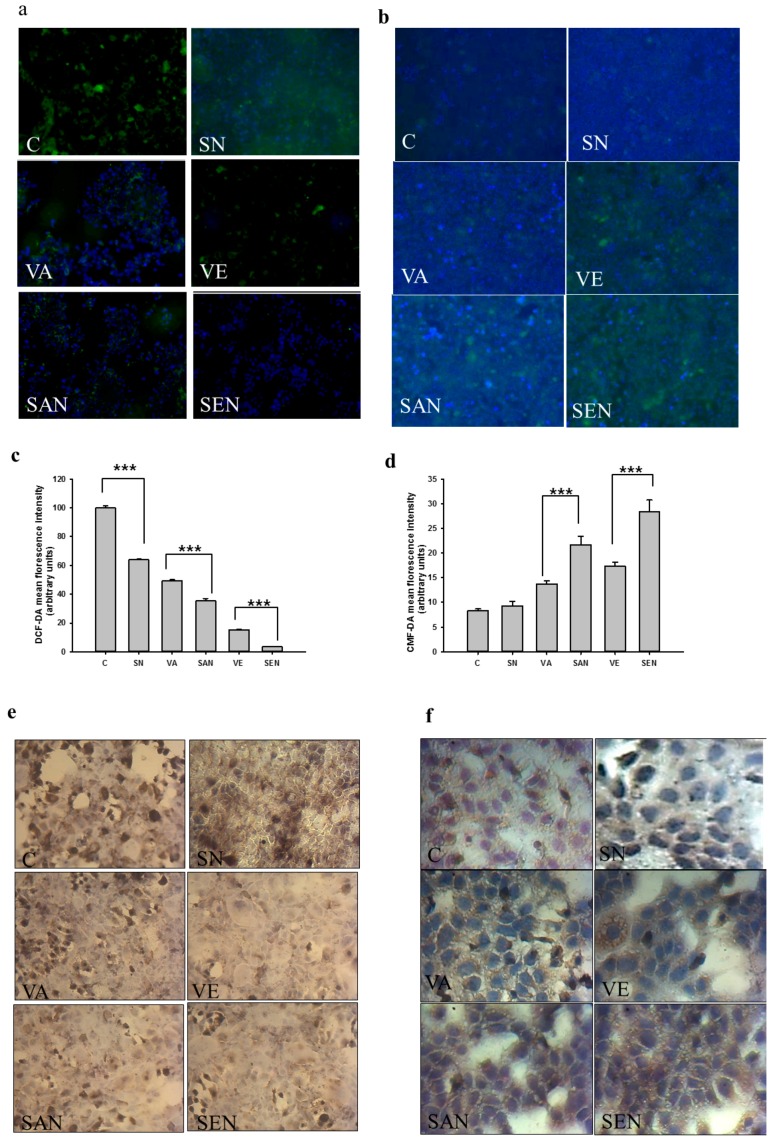
Effect of nanoemulsion on the cellular redox status of AML-12 cells exposed to H_2_O_2_. (**a**) Cells were co-treated with H_2_O_2_ and stained with DCF-DA; (**b**) The CMFDA fluorescence probe revealed GSH levels in AML-12 cells. Images were obtained under fluorescence microscopy from three separate experiments; (**c**) Quantification of DCF-DA fluorescence intensity using ImageJ software; (**d**) The mean fluorescence intensity of GSH was measured using ImageJ software; (**e**) GSSG expression in different groups was analyzed by immunocytochemistry. Images were obtained by light microscopy; (**f**) Effect of nanoemulsion on NADPH level was tested by immunocytochemistry. Images were obtained by light microscopy. C, control; SN, empty saponin nanoemulsion; VA, vitamin A; SAN, saponin nanoemulsion of vitamin A; VE, vitamin E; SEN, saponin nanoemulsion of vitamin E. All values are represented as mean ± SEM from three or more independent studies. *** *p* < 0.001.

To investigate whether the difference in hepatocyte viability after H_2_O_2_ and nanoemulsion exposure was correlated with ROS formation, we analyzed intracellular peroxide levels in AML-12 cells using the oxidant-sensitive probe DCF-DA with fluorescence microscopy. As shown in Figure 3a, H_2_O_2_ exposure increased DCF-DA fluorescence, which was reduced in cells treated with vitamin A and E. However, treatment with SEN and SAN resulted in comparatively lower levels of intracellular ROS (*p* < 0.001; Figure 3a,c). Cellular oxidative stress can also be measured by an alternative method, i.e., determining the intracellular level of reduced glutathione (GSH) concentration [33]. Cellular GSH levels, determined with the GSH–sensitive fluorescent dye CMFDA in AML-12 cells exposed to H_2_O_2_, were considerably depleted. However, GSH depletion in SAN and SEN cells was significantly mitigated compared with that in vitamin A, vitamin E, and SN cells (*p* < 0.001; Figure 3b,d). Under oxidative stress, GSH is oxidized to GSSG. We evaluated GSSG levels under same conditions to further confirm our results. When cells were exposed to H_2_O_2_, cellular GSSG was significantly higher in control cells, and it decreased more in SAN and SEN cells than in vitamin A and E cells (Figure 3e).

These data indicate that the GSSG level in nanoemulsion (SAN and SEN) cells was reduced efficiently compared with that in non-nanoemulsion (vitamin A and E) cells. During cellular defense against oxidative stress, NADPH is required for GSH generation and hence protects cells against oxidative damage [34]. Although the level of NADPH was reduced significantly in cells treated with H_2_O_2_ in our study, the decrease was less pronounced in non-emulsion cells than in vitamin A and E cells (Figure 3f).

### 2.6. Cellular Oxidative Damage

**Figure 4:**
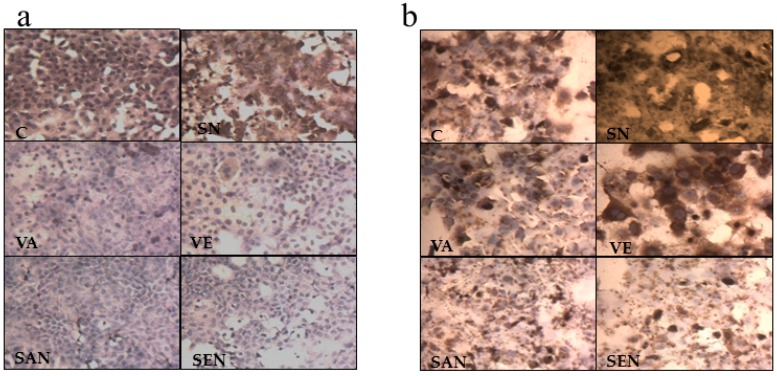

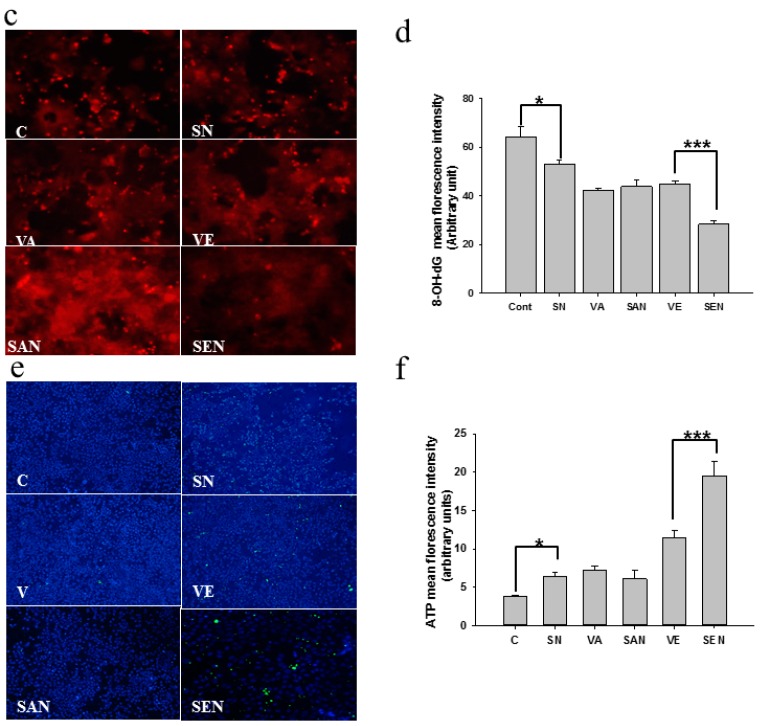
Protective effect of nanoemulsion against oxidative damage in AML-12 cells after H_2_O_2_ exposure. (**a**) Lipid peroxidation in AML-12 cells was detected with polyclonal anti-HNE antibody and DAB counterstain; (**b**) Protein carbonyl content of AML-12 cells was detected with DNP-specific antibody; (**c**) 8-OH-dG levels in AML-12 cells, which reflected the binding of avidin-TRITC, were analyzed using fluorescence microscopy; (**d**) The mean fluorescence intensity of avidin was quantified using ImageJ software; (**e**) Effect of nanoemulsion on intracellular ATP level was measured using FITC-labeled antibody; (**f**) ATP fluorescence intensity was quantified by ImageJ software. C, control; SN, empty saponin nanoemulsion; VA, vitamin A; SAN, saponin nanoemulsion of vitamin A; VE, vitamin E; SEN, saponin nanoemulsion of vitamin E. All values are represented as mean ± SEM from three or more independent studies. *** *p* < 0.001; * *p* < 0.05.

Oxidative DNA damage, protein oxidation, and lipid peroxidation are well-known markers that indicate cellular oxidative damage. An increase in cellular oxidative stress is directly proportional to lipid peroxidation, which leads to accumulation of potent cytotoxic lipid peroxides, such as HNE and MDA [35]. As shown in Figure 4a, the endogenous level of HNE adducts in proteins was significantly increased when AML-12 cells were exposed to H_2_O_2_, and the increase of lipid peroxidation was markedly reduced in emulsified vitamin samples compared with that in non-emulsified vitamin samples. For further investigation of whether emulsified vitamin treatment also decreased H_2_O_2_-induced protein damage, we evaluated protein oxidation by measuring the DNP-adduct of protein carbonyl content that corresponds to protein oxidation and hence protein damage after exposure to oxidative stress. As shown in Figure 4b, the endogenous level of carbonyl groups in proteins was significantly increased when cells were exposed to H_2_O_2_. Moreover, protein oxidation was reduced in SEN and SAN samples compared with that in SN samples.

8-OH-dG is an indicative marker for DNA damage [36] and measured specifically by fluorescence binding assay using avidin-conjugated TRITC [37]. Fluorescence intensity reflecting the endogenous level of 8-OH-dG was significantly increased in cells exposed to only H_2_O_2_. Conversely, the same dose of H_2_O_2_ in cells exposed to vitamins and especially nanoemulsion of vitamin E markedly protected cells from DNA damage (*p* < 0.001; Figure 4c,d). Oxidative stress induces cellular depletion of ATP levels [38]. In order to determine the ATP level of cells, FITC-linked ATP antibody was used. H_2_O_2_-induced cells showed a depletion of cellular ATP. The protective roles of SEN and SN against the loss of intracellular ATP levels were demonstrated; however, SAN did not demonstrate a protective effect (*p* < 0.001; Figure 4e,f).

## 3. Discussion

Oxidative stress induces ROS generation and, if not regulated, causes cellular damage by disrupting the oxidative status and redox potential of cells; it eventually leads to many pathological conditions that impede the proper functions of living systems. There are several natural compounds, including various vitamins, which help maintain the oxidative integrity of cells. Fat-soluble vitamins, such as vitamin A and E, can be incorporated into aqueous-based food via nano-encapsulation for effective delivery and good stability. This study was carried out to determine whether encapsulated vitamin A and E could provide additional health benefits. In the present study, we prepared nanoemulsions of vitamin A and E, using saponin as an emulsifier (Figure S1) and assessed stable emulsion formation by measuring the average particle size, droplet charge, polydispersity and emulsification. (Figure S2a,b)

Nanoemulsions did not induce toxicity in AML-12 cells (Figure S3a). AML-12 cells were used to examine the protective effects of SEN and SAN against oxidative stress-induced toxicity; H_2_O_2_ administration caused the generation of ROS, modulation of cellular redox status, and induction of cellular oxidative damage. Nanoemulsion exposure may have provided protective effects by increasing cell viability (Figure 1). SEN exhibited a significantly (*p* < 0.05) better ability to ameliorate oxidative stress-induced cellular damage compared with SAN and vitamins alone.

Both vitamin A and E have antioxidant activities, and their intracellular redox status and antioxidant defense system play a vital role against H_2_O_2_-induced cell death. To determine if the alleviation of H_2_O_2_-induced cytotoxicity was a consequence of enhanced nanoemulsion antioxidant activity, we further measured the antioxidant activity and redox status of cells. The CAA value of SEN was significantly higher than that of vitamin E, which was concomitant with the significant increase in the protective effect against H_2_O_2_-induced cytotoxicity (*p* < 0.05, Figure 2). We further investigated the intracellular redox status (GSH, GSSG, and NADPH) and oxidative damage (protein, DNA, and lipid damage) of cells to demonstrate that supplementation with emulsified vitamins provided more beneficial effects than supplementation with non-emulsified vitamins against oxidative stress-mediated cellular damage. ROS data support the hypothesis that saponin emulsification of vitamin A and E provides better protection (*p* < 0.001) against oxidative stress by decreasing the steady state of intracellular oxidants (Figure 3a,c). The initial cellular response to oxidative stress is reduced GSH level, which corresponds to an increase in oxidized GSH (GSSG) level [39,40] and affects cellular redox status; thus, we measured the GSH and GSSG levels of cells and found that they were in agreement with the ROS results (Figure 3b,d,e). These results were significant and indicated that SAN and SEN could scavenge ROS generation; SAN and SEN had more beneficial effects on the antioxidant system than their respective vitamins under oxidative stress conditions. SEN may have maintained the cellular redox balance by restoring cellular GSH and NADPH concentrations, shifting balance to the antioxidant condition.

Oxidative stress-induced ROS leads to DNA damage, protein carbonylation, and lipid peroxidation [41,42,43]. We also confirmed the protective effects of saponin-emulsified vitamin A and E against oxidative stress by demonstrating that the reactions leading to lipid peroxidation, protein carbonylation, and DNA damage were more efficiently suppressed by saponin-emulsified vitamins E (*p* < 0.001) than by non-emulsified vitamin. The results were consistent with the cellular redox status. SEN alleviated cellular damage (Figure 4a–c); however, it is interesting to note that SN also had a significant (*p* < 0.05) but lesser protective effect than SEN (*p* < 0.001). Oxidative damage eventually affects the mitochondria and results in depletion of cellular ATP levels, thus leading to energy impairment [38]. Cellular ATP level was restored when cells were treated with SEN and SN; *p* < 0.05 and 0.001 respectively (Figure 4e,f). It can be concluded that encapsulation improves the antioxidant activity of vitamin E against oxidative stress-induced cellular damage. The present study demonstrated for the first time that the saponin-based emulsification of vitamin A and E is a stable and efficient delivery system for fat-soluble vitamins, and also improves protective effect against oxidative stress-induced cellular damage. Furthermore, this emulsion system can be utilized for the fortification of beverages as it guarantees additional potential health benefits. This paper presents a new approach to determine the efficiency and potential of the nanoemulsion delivery system in terms of antioxidant activity for commercial food, beverage and cosmetic products.

## 4. Materials and Methods

### 4.1. Chemicals

Anti-human 4-hydroxynonenal (HNE)-Michael adduct antibody and anti-human dinitrophenyl (DNP) antibody were obtained from Calbiochem (La Jolla, CA, USA). DCF-DA (2,7-dichlorodihydrofluorescein diacetate) and MTT (3-(4,5-dimethylthiazol-2-yl)-2,5-diphenyltetrazolium bromide) were purchased from Sigma Chemical Co. (St. Louis, MO, USA). CellTracker Green 5-chloromethylfluorescein diacetate (CMFDA) was purchased from Molecular Probes (Life Technologies, Carlsbad, CA, USA). Saponin containing 8–25 *wt* % of sapogenin was purchased from Sigma–Aldrich Co. (St. Louis, MO, USA). Vitamin A (VA; minimum 1,000,000 IU vitamin A/g, containing 55.5% of vitamin A palmitate, 43.5% of peanut oil, and 1% of dl-α-tocopherol) and vitamin E-acetate (VE; 1100 IU vitamin E/g, containing 100% of dl-α-tocopherol) were purchased from DSM (Heerlen, The Netherlands). Double-distilled water was used for all solutions and emulsions.

### 4.2. Emulsion Preparation and Stability Testing

The schematic diagram for nanoemulsion preparation is shown in Figure S1. Nanoemulsions were prepared as described previously [29] with some modifications. Briefly, 10% (*w*/*w*) of the fat-soluble vitamins (A or E) and 1% (*w*/*w*) of natural surfactants (saponin) were blended in deionized water using a high-speed blender. Subsequently, the pre-emulsion mixture was passed through a high-pressure homogenizer (MN400BF, Micronox, Seongnam, Korea) at a pressure of 25 kpsi for seven cycles to produce nanoemulsions. The particle size and zeta potential of nanoemulsions were determined by photon correlation spectroscopy (Malvern Nano ZS90, Malvern, UK) as described previously [44]. 10% (*w*/*w*) of the fat-soluble vitamins (VA or VE) and 1% of the natural surfactants (lecithin and saponin) were emulsified using the high-speed blender or the high-pressure homogenizer in the deionized water to determine the emulsification index (EI_24h_). Then, mixtures were transferred to a mass cylinder and stored at ambient temperature for 24 h to measure EI_24h_. EI_24h_ is calculated as percentage of height of emulsified layer from height of the total mixture.

### 4.3. Cell Culture

Alpha mouse liver 12 (AML-12) cells were obtained from the American Type Culture Collection (ATCC, CRL-2254). Cells were cultured in a 90% 1:1 mixture of Dulbecco’s modified Eagle’s medium (DMEM) and DMEM/F-12 (supplemented with 0.005 mg/mL insulin, 0.005 mg/mL transferrin, 5 ng/mL selenium, and 40 ng/mL dexamethasone) and 10% fetal bovine serum, at 37 °C in a 5% CO_2_ humidified incubator.

### 4.4. Cell Viability

AML-12 cells were seeded in 96-well culture plates at a density of 1 × 10^5^ cells/mL overnight. The media was removed after incubation, and cells were washed with PBS and treated with a variable dose of emulsified and non-emulsified samples for 24 h. vitamins were dissolved in 100% ethanol as stock solution and further dissolved in growth medium for treatment. Ethanol concentration was kept at 0.001% for all cell treatment conditions.Following incubation for 3 h with 0.5 mg/mL of MTT, cell viability was measured by assessing the catalytic ability of mitochondrial dehydrogenases that convert MTT to blue-colored formazan salts. The concentration of formazan dissolved in DMSO was measured (OD 595 nm) using a microplate reader after 3 h of incubation at 37 °C.

### 4.5. Cellular Antioxidant Activity (CAA) Assay

CAA assay was performed as described previously [32]. Briefly, HepG-2 cells were seeded at density of 5 × 10^4^/ well in 96-well culture plates. After 24 h of incubation, cells were washed with PBS and treated with 25 μM DCF-DA in the growth medium with or without emulsified and non-emulsified samples for 1 h. After incubation, 100 μL of HBSS containing 600 μM of ABAP was added in each well, except for blank that was treated with only 100 μL of HBSS, and placed in a multi-mode microplate reader at 37 °C with emission and excitation wavelengths of 538 nm and 485 nm, respectively. The fluorescence values were measured for 12 cycles at every 5 min interval. Samples, control, and blank were analyzed in triplicate. After subtraction of the blank fluorescence reading, the area under the curve for fluorescence versus time was integrated to calculate the CAA value of each sample at the same concentration by the following equation:

CAA unit = 100 − (∫SA/∫CA) × 100

whereas ∫SA and ∫CA are integrated under the fluorescence versus time curve of test sample and control, respectively.

### 4.6. Immunohistochemistry

For immunohistochemistry, AML-12 cells were seeded on glass coverslips and incubated 37 °C until 90% confluent. After incubation, cells were treated with H_2_O_2_ and emulsified and non-emulsified samples for 2 h. Coverslips were washed twice with PBS, and cells were fixed with 4% paraformaldehyde for 10 min. Immunocytochemistry stainings for 4-HNE, NADPH, GSSG, and DNP were performed according to manufacturer’s instructions as described previously [45] with some modifications. Briefly, endogenous peroxidase was inhibited after fixation by incubating cells in 0.3% H_2_O_2_ with PBS for 5 min at 20 °C. Cells were washed three times with PBS for 5 min each time. Nonspecific absorption was minimized by incubating the coverslips with 6% bovine serum albumin (BSA) for 1 h at room temperature and overnight incubation with 1:200–1:500 dilution of primary antibodies at 4 °C. After washing with PBS three times for 5 min each time, cover slips were incubated with specific secondary antibodies (post-primary) for 30 min at room temperature. All treatment groups were incubated under the same conditions with the same concentration of antibodies and at the same time; thus, the immunostainings were comparable among different treatment groups.

### 4.7. Fluorescence Microscopy

Intracellular ROS production was measured using the oxidant-sensitive fluorescent probe DCF-DA with fluorescence microscopy. Cells were grown at 2 × 10^6^ cells per 100 mm plate containing glass slides coated with poly-l-lysine and maintained in the growth medium for 24 h. Cells were treated with 10 μm DCF-DA for 15 min and washed with PBS, and a cover glass was placed on the glass slide. DCF-DA fluorescence (excitation, 488 nm; emission, 520 nm) was imaged under Axio Vert. A1 microscope. Intracellular GSH level was also determined by using the GSH-sensitive fluorescent dye CMFDA. AML-12 cells (1 × 10^5^ cells/mL) were incubated with 5 μm CellTracker Green CMFDA for 30 min. The fluorescence caused by conjugation of GSH with CMFDA CellTracker was analyzed by the Axio Vert. A1 microscope. Levels of 8-OH-dG and ATP in AML-12 cells were estimated by fluorescence binding assay. After exposure of AML-12 cells to oxidative stress, cells were fixed and permeabilized with ice-cold methanol for 15 min. DNA damage was visualized with avidin-conjugated TRITC (1:200 dilution). For ATP detection, FITC-linked ATP antibody was used for fluorescence microscopy with a 540-nm excitation and 588-nm emission.

### 4.8. Statistical Analysis

All studies were replicated three times with representative data shown as mean ± SEM. Fluorescence intensity was quantified by ImageJ software. Statistical analysis was performed by the Student’s *t*-test. A value of *p* < 0.05 was considered significant difference.

## Supplementary Materials

Supplementary materials can be found at www.mdpi.com/1422-0067/17/9/1406/s1.
