# Supplementary material for: Saponin-Based Nanoemulsification Improves the Antioxidant Properties of Vitamin A and E in AML-12 Cells
Source: Int J Mol Sci. 2016 Aug 26;17(9):1406. doi: 10.3390/ijms17091406 (PMC5037686; doi:10.3390/ijms17091406)
Supplement: Supplementary file 1 [file ijms-17-01406-s001.pdf]

Supplementary Materials: Saponin-Based Nanoemulsification Improves the Antioxidant Properties of Vitamin A and E in AML-12 Cells

Qaisra Naheed Choudhry, Mi Jeong Kim, Tae Gyun Kim, Jeong Hoon Pan, Jun Ho Kim, Sung Jin Park, Jin Hyup Lee and Young Jun Kim

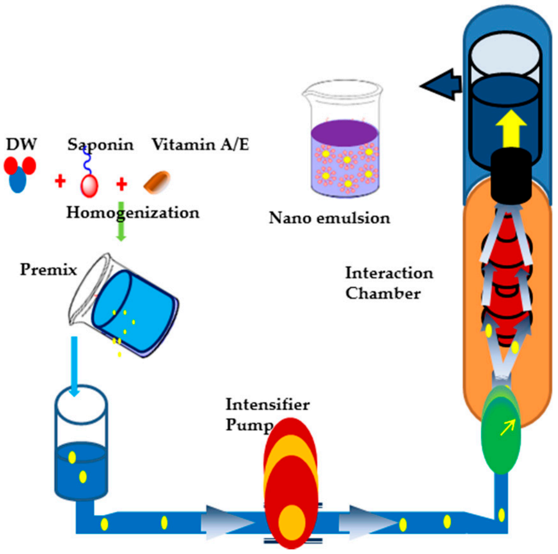

Figure S1. Schematic flow diagram representing the preparation method of o/w nanoemulsions of vitamin A and E using saponin as surfactant. 10% (*w*/*w*) of fat-soluble vitamins (VA or VE) and 1% (*w*/*w*) of natural surfactants (saponin) were blended in deionized water using a high-speed blender. Subsequently, the pre-emulsion mixture was passed through a high-pressure homogenizer (MN400BF, Micronox, Seongnam, Korea) at a pressure of 25 kpsi for seven cycles to produce nanoemulsions.

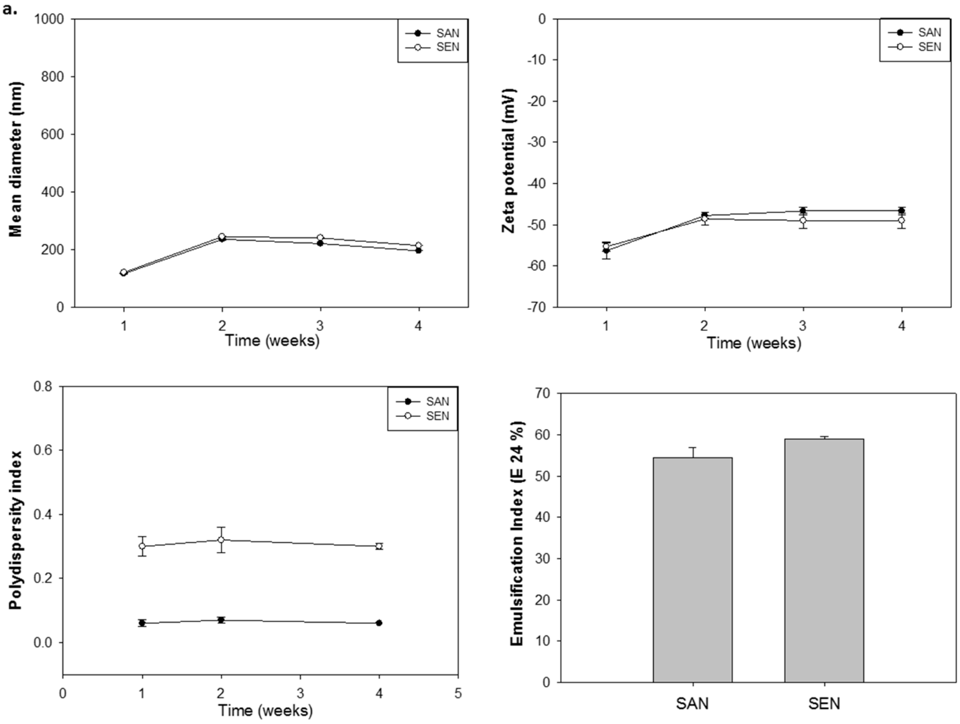

Figure S2. *Cont.*

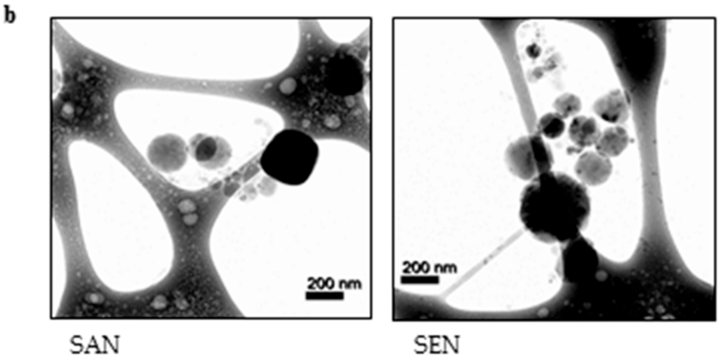

Figure S2. Nanoemulsion characterization. (**a**) Physiochemical properties of SAN and SEN; (**b**) Cryo-TEM images of SAN and SEN. SAN, saponin nanoemulsion of vitamin A; SEN, saponin nanoemulsion of vitamin E.

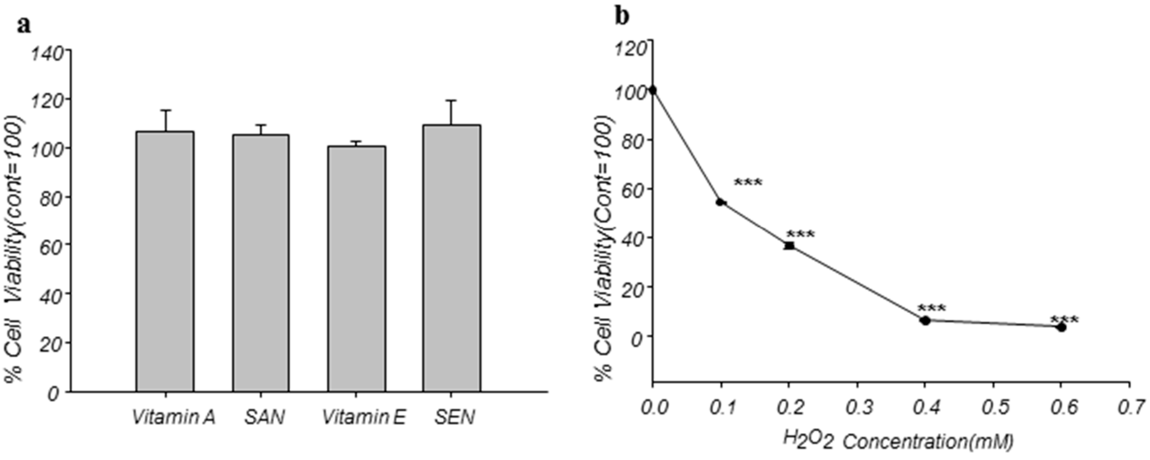

Figure S3. Cytotoxicity. (**a**) Cells were exposed to 200 μg/mL of Vitamin A, SAN, vitamin E, and SEN for 16 h. Cell viability was determined by MTT assay; (**b**) AML-12 cells were exposed to exogenous H_2O_2 at the indicated concentrations for 16 h. Cell viability was determined by MTT assay. SAN, saponin nanoemulsion of vitamin A; SEN, saponin nanoemulsion of vitamin E. *** $p < 0.001$.
